# Scope, Prospects, and Limitations of Living Systematic Reviews in Medical Literature: A Narrative Overview

**DOI:** 10.7759/cureus.112631

**Published:** 2026-07-14

**Authors:** Charanya Natarajan, G Harshini, Gomathi T Priyadharsini, Thangadurai Maheswaran, N. S Naveenraj, Malliga Venkat

**Affiliations:** 1 Anatomy, Shri Sathya Sai Medical College and Research Institute, Sri Balaji Vidyapeeth (Deemed to be University), Puducherry, IND; 2 Oral and Maxillofacial Pathology, Sri Ramakrishna Dental College and Hospital, Coimbatore, IND; 3 Anatomy, Vivekanandha Dental College for Women, Namakkal, IND; 4 Oral and Maxillofacial Pathology, Adhiparasakthi Dental College and Hospital, Melmaruvathur, IND; 5 Public Health Dentistry, Adhiparasakthi Dental College and Hospital, Melmaruvathur, IND

**Keywords:** artificial intelligence, clinical practice guideline, evidence-based medicine, evidence synthesis, literature review, living systematic review, practice guidelines, research design, systematic review and meta analysis

## Abstract

Living systematic reviews (LSRs) have emerged as a response to the persistent lag between the publication of primary research and its incorporation into systematic reviews, a delay that has historically spanned several years and can render a meaningful proportion of reviews outdated within two years of publication. Since their formal introduction in 2014, LSRs have evolved from a single conceptual proposal into a maturing methodological field, supported by a dedicated PRISMA reporting extension, decision frameworks for determining when a living approach is warranted, and demonstrated applications in COVID-19 therapeutics, vaccine effectiveness, long COVID, and the development of formal clinical guidelines. Uptake accelerated markedly during the COVID-19 pandemic, and automation, ranging from human-machine collaborative workflows to large language models, has become central to sustaining the frequency of updates required by the living model, with reported gains in screening and extraction efficiency. However, methodological guidance for reporting and appraising LSRs remains underdeveloped relative to the pace of adoption, statistical methods for repeated meta-analytic updating are heterogeneous and imperfectly matched to network meta-analyses, and operational surveys consistently document inconsistent update schedules, limited communication of living status, and resource constraints that threaten their long-term sustainability. Realizing the full clinical and policy value of LSRs will require coordinated progress across methodological guidance, automation, and institutional support. Hence, this narrative review aims to examine the above concerns related to LSR.

## Introduction and background

Systematic reviews are widely regarded as the most reliable form of evidence synthesis for clinical and policy decision-making; however, their value is undermined when the underlying evidence base moves faster than the review process can accommodate [1]. Primary study results typically take between 2.5 and 6.5 years to be incorporated into a published systematic review, and within two years of publication, 23% of reviews fail to capture evidence capable of changing their conclusions on the effectiveness or harms of an intervention [1]. Living systematic reviews (LSRs) were proposed in 2014 as a direct response to this evidence-practice gap, combining ongoing surveillance of the literature with continual updating to maintain a current, high-quality synthesis [1]. The uptake of the concept accelerated sharply after 2020, driven substantially by the COVID-19 pandemic, with the approach subsequently extended from its origins in trauma and oncology research to vaccine effectiveness, long COVID therapeutics, and formal clinical guideline development [2,3].

Sustaining an LSR depends on combining human judgment with automation across searching, screening, data extraction, and risk-of-bias assessment, as human effort alone cannot support the frequency of updating that a living review demands [4]. Artificial intelligence (AI) and, more recently, large language models (LLMs) have been applied across these tasks with reported gains in recall, accuracy, and workload reduction, positioning automation as a central determinant of whether the living model can be sustained at scale [4,5]. However, methodological guidance has not kept pace with this practical expansion: a scoping review of the guidance literature found only 21 eligible papers addressing how LSRs should be conducted, reported, published, and appraised, with substantial gaps in guidance for reporting results and assessing review quality [6]. Methodological surveys of the published LSR literature have similarly found inconsistent updating practices, limited communication of a review's living status, and reviews that persist in name without receiving further updates [7].

Despite the rapid proliferation of LSRs across clinical fields and the parallel growth of automation tools, relatively few recent narrative overviews have integrated the conceptual foundations, clinical applications, technological developments, and methodological challenges of LSRs in a single article. This narrative overview was based on the literature identified through PubMed searches using keywords related to LSRs, living evidence synthesis, living guidelines, automation, AI, and evidence synthesis. English-language articles published in the past decade were considered. References were selected based on author judgment, with priority given to landmark publications, recent reviews, clinical studies, methodological guidance, and clinical guidelines relevant to the scope of this review. No formal systematic search protocol or risk-of-bias assessment was performed. This narrative overview addresses this gap by providing an interpretive synthesis of the conceptual foundations, clinical applications, automation, and methodological challenges of LSR.

## Review

Concept and definitional scope

LSRs were conceived as a structural response to the chronic problem of evidentiary obsolescence in conventional systematic review. Elliott et al. proposed the concept after documenting that primary study results typically take 2.5 to 6.5 years to reach incorporation into a published systematic review, and that within two years of publication, 23% of reviews would already have failed to reflect evidence capable of altering conclusions on efficacy or harm [1]. An LSR is a high-quality, continuously updated online summary of evidence that is refreshed as new research emerges, made feasible by gains in production efficiency and closer adherence to the norms of scholarly communication [1]. This framing positioned the LSR not as a replacement for a standard systematic review but as a complementary contribution to a broader, technology-enabled evidence ecosystem [1]. LSRs should be distinguished from updated systematic reviews, which undergo periodic revisions without continuous surveillance; rapid reviews, which prioritize timeliness over comprehensive updating; living guidelines, which use continuously updated evidence to inform recommendations; and living network meta-analyses, which extend the living approach to comparative analyses across multiple interventions.

Bibliometric mapping of the field shows that uptake was initially slow, with relatively few LSR-related publications appearing between 2014 and 2019, before an explosive rise in output during 2020 and 2021, coinciding with the COVID-19 pandemic [2]. The approach was first operationalized outside the original proposal in the Collaborative European NeuroTrauma Effectiveness Research in Traumatic Brain Injury (CENTER-TBI) program, followed by the articulation of a living network meta-analysis framework in 2016 and the launch of the Cochrane Living Evidence Network in the same year, which consolidated methodological resources and dissemination infrastructure for the field [2]. The World Health Organization adopted living guidance for maternal and perinatal health in 2019, and the first COVID-19-specific LSR appeared in April 2020, after which the scope of application expanded rapidly across clinical and public health domains [2]. Despite this growth, more than half of the identified LSR series had not been updated at the time of assessment, and bibliometric evidence points to a pressing need for methodological standardization across the expanding literature [2]. These observations also suggest that the designation "living" should ideally reflect ongoing evidence surveillance, predefined update processes, and continued implementation of planned updates rather than the label alone.

This need has only been partially addressed in the literature. A scoping review of methodological guidance identified only 21 eligible papers addressing the conduct, reporting, publishing, and appraisal of LSRs, with guidance concentrated heavily on conducting and publishing procedures, while reporting of results, discussion, and certainty of evidence, and criteria for appraising LSR quality remained substantially underdeveloped [6]. This evidence gap informed the subsequent development of the PRISMA-LSR extension, an add-on to the PRISMA 2020 statement comprising a dedicated checklist, tailored flow diagram, and explicit recommendations for reporting the living status of a review, intended to standardize the transparent and accurate communication of LSR conduct for authors, editors, peer reviewers, and end users [8]. Together, these developments delineate the scope of the LSR concept: a methodologically rigorous, iteratively updated evidence synthesis format whose formal reporting and appraisal infrastructure is still consolidating, even as its practical uptake accelerates.

Role, relevance, and clinical applications

The clinical and policy relevance of LSRs is most evident in their capacity to maintain the currency of clinical guidelines, policy recommendations, and other evidence-informed decision-making resources. Living guidelines have been defined as an optimization of the standard guideline development process that allows individual recommendations, rather than an entire document, to be updated as soon as pertinent new evidence emerges [9]. Realizing this model depends on close collaboration between LSR teams and guideline panels, agreed thresholds for triggering a change in recommendation, and workable approaches to publication and dissemination, with the sustainability of living guidance ultimately resting on the sustainability of its underlying LSR [9]. A subsequently proposed operational framework organizes the development of living guidelines into five domains: planning, production, reporting, dissemination, and implementation, drawing on the Grading of Recommendations Assessment, Development and Evaluation (GRADE) Evidence-to-Decision approach and incorporating AI to support continuous literature monitoring and data extraction, alongside explicit criteria for transitioning between living and standard recommendation modes [3]. Transparent reporting of updates and structured external reviews were identified as central to sustaining the trustworthiness of living guidance over time [3].

Not every question merits a living approach, and criteria have been proposed to guide the decision-making process. Drawing on the experience of developing living reviews on COVID-19 vaccine effectiveness for the German Standing Committee on Vaccination, a needs-and-feasibility framework was developed comprising criteria such as the relevance of the question, the baseline certainty of evidence, the expected frequency of new evidence and its likely impact on that certainty, and the availability of sufficient human resources and dissemination capacity [10]. This decision framework illustrates that the relevance of the LSR approach is contingent on topic-level characteristics rather than being universally applicable [10].

The pandemic provided the clearest large-scale demonstration of the relevance of the LSR in practice. The Pan American Health Organization's living review of COVID-19 therapeutics grew from an initial 18 interventions and 44 studies in April 2020 to 305 interventions and 924 randomized controlled trials after 48 updates, identifying interventions that demonstrated benefit within the available evidence at successive review updates while recognizing that the effectiveness of some therapies, particularly neutralizing monoclonal antibodies, has evolved with changing viral variants, clinical settings, and treatment indications [11]. A parallel three-way collaboration between the Australian Living Evidence Collaboration, Bond University, and the Epistemonikos COVID-19 L·OVE (Living OVerview of Evidence) platform built a combined evidence base of 218 randomized trials and 56 systematic reviews on long COVID therapeutics, which was later refined to 102 trials under a harmonized eligibility definition, with only approximately a quarter of trials identified independently by all three search strategies, underscoring the value of complementary search approaches for sustaining a living evidence base [12]. However, the uptake of the model has not guaranteed its faithful execution: an assessment of Cochrane's 25 COVID-19 living reviews found that just over half were never updated after the initial publication, and only four were updated more than once, with authors reporting that the inability to communicate a review's living status to readers was a recurrent source of frustration [13]. These contrasting experiences indicate that the relevance of LSRs to clinical and policy decision-making is realized only when institutional and editorial support matches the ambition of the living format [11,13].

Automation and technological prospects

Automation has become central to discussions on how LSRs can remain feasible at a large scale. An early conceptual paper argued that human effort, a scarce resource best reserved for tasks that automation cannot reliably perform, could be combined with machine automation across searching, eligibility screening, full-text retrieval, data extraction, and risk-of-bias assessment, with each element reinforcing the other to expand review productivity beyond what either could achieve alone [4]. This human-machine partnership framing anticipated much of the subsequent technological development in the field and was explicitly noted to apply equally to conventional systematic reviews [4].

A synthesis of studies applying AI within living evidence workflows found that of 24 eligible studies, 34 distinct AI or semi-automated tools had been deployed, with the majority directed at data extraction and risk-of-bias assessment rather than at the publication-update phase, where only a single tool had been applied [5]. Across these heterogeneous studies evaluating different review tasks, datasets, and AI tools, descriptive performance measures included a mean reported recall of 96.24% and a mean F1-score of 92.17%; these figures should not be interpreted as universally applicable estimates of AI performance [5]. This body of evidence flagged persistent challenges, including inconsistent precision across topics, the risk of selection bias arising from skewed training data, data protection concerns linked to sensitive research content, and practical costs of implementation and maintenance [5].

LLMs have extended this trajectory to tasks that were previously considered resistant to automation. A collaborative two-model workflow using GPT-4-turbo and Claude-3-Opus for structured data extraction achieved 87% concordance between models in a held-out test set, with concordant responses reaching 0.94 accuracy; when discordant outputs were cross-critiqued between the two models, accuracy on those cases rose from approximately 0.4-0.5 to 0.76 [14]. In title and abstract screening, refined GPT-4o prompts achieved 100% sensitivity for studies ultimately confirmed eligible after full-text review, although sensitivity for the initial title-and-abstract judgments made by human reviewers ranged more widely, from 58% to 100% across successive updates, alongside simulated workload reductions of 65% to 85% [15]. A purpose-built platform for accelerating screening, evaluated across seven reviews, required manual screening of only 36% of records on average before reaching its stopping criterion, achieved a mean work-saved-over-sampling value of 59% at 95% recall, and misclassified none of a sampled set of rejected records, translating into an estimated saving of approximately seven weeks of screening time for a review of 10,000 records [16]. Table 1 summarizes the reported performances of these automation approaches across the principal review phases. Despite these promising results, current LLMs remain susceptible to hallucination, prompt-dependent variability, limited reproducibility, evolving model architectures, and data privacy considerations, and therefore continue to require human oversight and verification.

**Table 1:** Reported performance of automation and artificial intelligence tools across living systematic review phases LLM, large language model Source: Original table created by the authors based on data synthesized from references [4,5,14-16].

Tool or approach	Review phase	Reported performance
Human-machine collaborative workflows	Searching, screening, extraction, risk-of-bias assessment	Combines human and automated effort to expand productivity; performance is task-dependent [4]
Mixed AI/semi-automated tools (34 tools across 24 studies)	Predominantly data extraction and risk-of-bias assessment; rarely applied to publication updating	Mean recall 96.24%; mean F1-score 92.17%; precision 0.2-100% [5]
Collaborative LLMs (GPT-4-turbo and Claude-3-Opus)	Structured data extraction	87% inter-model concordance; accuracy 0.94 for concordant items, rising to 0.76 after cross-critique of discordant items [14]
GPT-4o screening prompts	Title/abstract screening	100% sensitivity for eventually-included studies; 65-85% simulated workload reduction [15]
LitQuest screening platform	Title/abstract screening	Mean 36% of records required manual screening; work saved over sampling 59% at 95% recall; 0% misclassification in sampled audit [16]

These efficiency gains have not yet been fully extended to synthesis and publication updating, which remain comparatively underserved by automation and continue to raise the methodological and operational challenges discussed below.

Methodological and statistical challenges

Updating meta-analyses repeatedly within a living framework introduces statistical challenges that are largely absent from static syntheses. Repeated interim analyses using conventional meta-analytic methods inflate the probability of falsely concluding that an effect is statistically significant, and inaccurate estimation of between-study heterogeneity exacerbates this risk. Four candidate solutions, namely the law of the iterated logarithm, Shuster method, trial sequential analysis, and sequential meta-analysis, have been proposed, of which the latter two also address the risk of failing to detect a genuine effect and account for heterogeneity [17]. These approaches are primarily intended to reduce the risk of incorrect conclusions arising from repeated statistical testing during successive review updates. Their relevance is greatest when living reviews directly inform formal recommendations or decision-making rather than simply maintaining an up-to-date evidence summary. A subsequent scoping review mapping methods for updating pairwise and network meta-analyses across 51 eligible studies found that trial sequential analysis was by far the most frequently applied approach, that the overwhelming majority of methodological work addressed pairwise rather than network meta-analysis, and that no single method demonstrated superiority across contexts, leaving network meta-analysis updating comparatively underserved by dedicated statistical guidance [18].

Whether these statistical corrections are required depends on the review purpose. Adjustment for multiplicity was judged unnecessary when an LSR was intended simply to provide the best current evidence without a formal decision-triggering mechanism, but potentially important when a review operated under a protocol involving a pre-specified stopping rule, such as in living guideline development or reimbursement decisions, a distinction that existing multiplicity-adjustment methods do not always make explicit [19].

Methodological choices beyond statistics also shape evidence reliability. Within a living Cochrane network meta-analysis of systemic psoriasis treatments, transitioning from the original risk-of-bias tool to its revised version yielded only fair-to-moderate inter-rater agreement and prompted substantial reclassification of individual trials, with 45% of trials assessed for the Psoriasis Area and Severity Index 90 (PASI 90) outcome and 60% of trials assessed for serious adverse events changing risk-of-bias category; the resulting sensitivity analyses showed a modest effect on efficacy estimates but a more pronounced effect on estimates for serious adverse events [20]. Similarly, within a living network meta-analysis of COVID-19 treatments, incorporating conference abstracts of variable reporting quality, only 7% of which met high reporting standards, enabled meta-analytic pooling at time points that would otherwise have lacked sufficient data, but altered effect estimates in over half of the comparisons and imprecision ratings in one-sixth of cases once minimally important difference thresholds were applied [21]. These findings collectively indicate that the methodological instruments underpinning meta-analytic updating, from statistical stopping rules to risk-of-bias assessment tools and inclusion criteria for evidence sources, materially influence the conclusions a living review reaches at each iteration [20,21].

Operational limitations and sustainability challenges

Even when the methodological case for a living approach is accepted, sustaining the practice has proven difficult. A mixed-methods evaluation of pilot LSRs found that the workload involved varied enormously between teams, with monthly citation-screening volumes ranging from 3 to 300 records and author time commitment ranging from 5 minutes to 32 hours per month. This wide variation likely reflects differences in the review scope, literature volume, update frequency, automation support, number of outcomes assessed, and available team resources. While enthusiastic, participants consistently identified constraints in the publication process, ongoing workload management, and the absence of resources to sustain reviews over the long term [22]. A methodological survey of 76 eligible LSRs comprising 279 published versions found a median of only two versions per review and also reported that among reviews with a pre-specified update schedule, 42% had exceeded three times their planned interval since the last update, while none of the reviews surveyed included a retirement notice signaling withdrawal from the living status [7]. A subsequent update to this survey, covering 168 LSRs, including 92 newly identified since the original assessment, confirmed continued rapid growth in LSR numbers but again highlighted highly variable approaches to update timing, limited use of interactive dissemination platforms, and persistent methodological limitations requiring more explicit, pre-specified updating strategies [23]. These patterns were echoed in Cochrane's COVID-19 living reviews; over half of them were never updated after the initial publication despite an ongoing pandemic evidence base [13].

These operational strains sit within a wider set of concerns about systematic review quality that are not unique to the living format, but a living review of the meta-research literature itself has continued to be tracked across successive updates. The aforementioned living meta-research review has now catalogued 69 recurring problems affecting systematic reviews broadly, including critically low methodological quality, absent protocols, spin in abstracts, and unresolved conflicts of interest, alongside persistent geographic concentration of both review authorship and included primary research in high-income countries despite modest improvements in gender diversity [24]. Taken together, this evidence indicates that the principal limitations of LSRs are less a matter of unproven concepts than of sustained execution: teams frequently lack the durable resourcing, dissemination infrastructure, and transparent exit criteria needed to keep a living review both current and clearly communicated to its users over its full life cycle [7,22]. Potential retirement criteria include stabilization of the evidence base, prolonged absence of clinically important new studies, declining clinical priority, withdrawal of funding or review teams, and transition of the evidence into established clinical guideline programs.

## Conclusions

LSRs represent a substantive methodological advance over static systematic reviews, offering a mechanism to narrow the gap between the pace of primary research and the currency of synthesized evidence underpinning clinical and policy decisions. The concept has matured from a single proposal into a body of reporting guidance, decision criteria for when a living approach is warranted, and a growing evidence base demonstrating its value in fast-moving fields such as COVID-19 therapeutics, vaccine effectiveness, and long COVID. Automation and increasingly LLMs are reshaping the feasibility of maintaining these reviews, delivering measurable gains in screening and extraction efficiency, although publication updating remains comparatively unautomated. Simultaneously, the statistical demands of repeated meta-analytic updating, the sensitivity of conclusions to risk-of-bias assessment tools and evidence-inclusion decisions, and the practical burden of sustaining review teams over years rather than months remain substantial and only partially resolved issues. For LSRs to fulfil their promise at scale, methodological guidance, automation tools, and institutional resourcing need to advance together, with particular attention to the transparent communication of a review's living status, pre-specified retirement criteria, and equitable global participation in both conducting and benefiting from continuously updated evidence. As a narrative overview, this article provides an interpretive synthesis of selected literature rather than a comprehensive systematic evidence synthesis. Although recent landmark publications and methodological developments were prioritized, the absence of a formal systematic search strategy and quality appraisal may have resulted in omission of some relevant studies.
